# QuickStats

**Published:** 2013-03-15

**Authors:** Debra Blackwell, Tainya C. Clarke

**Figure:**
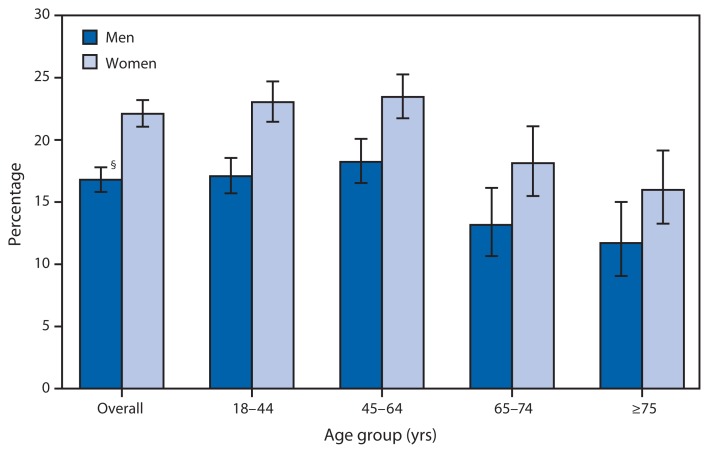
Percentage of Adults Aged ≥18 Years Who Often Felt Worried, Nervous, or Anxious,^*^ by Sex and Age Group — National Health Interview Survey, United States, 2010–2011^†^ ^*^Based on a survey question that asked respondents, “How often do you feel worried, nervous, or anxious? Would you say daily, weekly, monthly, a few times a year, or never?” Persons reporting daily or weekly feelings of worry, nervousness, or anxiety were categorized as often worried, nervous, or anxious. Unknowns were not included in the denominators when calculating percentages. ^†^Estimates are based on household interviews of a sample of the U.S. civilian, noninstitutionalized population. ^§^95% confidence interval.

During 2010–2011, women (22.1%) were more likely than men (16.8%) to often feel worried, nervous, or anxious. Among men, those aged 45–64 years were about as likely (18.2%) as men aged 18–44 years (17.1%) but more likely than men aged 65–74 years (13.2%) and ≥75 years (11.7%) to often have feelings of worry, nervousness, or anxiety. Women aged 18–44 years were about as likely (23.0%) as women aged 45–64 years (23.5%) but more likely than women aged 65–74 years (18.1%) and women aged ≥75 years (16.0%) to often feel worried, nervous, or anxious.

**Source:** National Health Interview Survey, 2010 Quality of Life and 2011 Functioning and Disability supplements. Data were colleccted from a subset of the adults randomly selected for the sample adult component of the NHIS questionnaire. Available at http://www.cdc.gov/nchs/nhis.htm.

